# *In vitro* antibacterial activity of ZnO and Nd doped ZnO nanoparticles against ESBL producing *Escherichia coli* and *Klebsiella pneumoniae*

**DOI:** 10.1038/srep24312

**Published:** 2016-04-13

**Authors:** Abdulrahman Syedahamed Haja Hameed, Chandrasekaran Karthikeyan, Abdulazees Parveez Ahamed, Nooruddin Thajuddin, Naiyf S. Alharbi, Sulaiman Ali Alharbi, Ganasan Ravi

**Affiliations:** 1 PG and Research Department of Physics, Jamal Mohamed College, Tiruchirappalli-620020, Tamil Nadu, India; 2 Division of Microbial Biodiversity and Bioenergy, Department of Microbiology, Bharathidasan University, Tiruchirappalli-600024, Tamil Nadu, India; 3 Department of Botany and Microbiology, College of Science, King Saud University, Riyadh-11451, Kingdom of Saudi Arabia; 4 School of Physics, Alagappa University, Karaikudi-630004, Tamil Nadu, India

## Abstract

Pure ZnO and Neodymium (Nd) doped ZnO nanoparticles (NPs) were synthesized by the co-precipitation method. The synthesized nanoparticles retained the wurtzite hexagonal structure. From FESEM studies, ZnO and Nd doped ZnO NPs showed nanorod and nanoflower like morphology respectively. The FT-IR spectra confirmed the Zn-O stretching bands at 422 and 451 cm^−1^ for ZnO and Nd doped ZnO NPs respectively. From the UV-VIS spectroscopic measurement, the excitonic peaks were found around 373 nm and 380 nm for the respective samples. The photoluminescence measurements revealed that the broad emission was composed of ten different bands due to zinc vacancies, oxygen vacancies and surface defects. The antibacterial studies performed against extended spectrum β-lactamases (ESBLs) producing strains of *Escherichia coli* and *Klebsiella pneumoniae* showed that the Nd doped ZnO NPs possessed a greater antibacterial effect than the pure ZnO NPs. From confocal laser scanning microscopic (CLSM) analysis, the apoptotic nature of the cells was confirmed by the cell shrinkage, disorganization of cell wall and cell membrane and dead cell of the bacteria. SEM analysis revealed the existence of bacterial loss of viability due to an impairment of cell membrane integrity, which was highly consistent with the damage of cell walls.

Because of many biological processes taking place at the nanoscale level, there is the potential that engineered nanomaterials may interact with biomolecules and cellular processes^1^. ZnO nanoparticles (NPs) are believed to be nontoxic, biosafe and biocompatible^2^. They have also been used as drug carriers, in cosmetics and fillings in medical materials^3^,^4^. The modification of metal oxide nanoparticles by doping or substituting with special atom(s) gives a possibility to improve the electrical and optical properties of materials by changing the surface properties. Therefore, such systems are becoming more and more important in materials science and being used as photo-catalysts, solar cells and gas sensors^5^,^6^,^7^,^8^,^9^. There are several methods reported in the literature for the synthesis of undoped and doped ZnO nanoparticles which can be categorized into either chemical or physical methods^10^,^11^ such as sol-gel method^12^, solvothermal^13^ and co-precipitation method^14^. Among the various methods, co-precipitation is one of the most important methods to prepare the nanoparticles. The co-precipitation method reduces the temperature of the reaction where a homogeneous mixture of reagent precipitates. It is a simple method for the synthesis of nanopowders of metaloxides, which are highly reactive in low temperature sintering. In the literature, it has been reported that a suitable Nd concentration can improve the blood compatibility and excellent hemocompatibility of ZnO thin-films due to the hydrophobic surface and the anticoagulant property of the rare earth elements^15^.

Metal oxide nanoparticles have been studied extensively to explore their utility as a potential antibacterial agent^16^,^17^. The deposition of the metal oxide nanoparticles on the surface of bacteria or accumulation of nanoparticles either in the cytoplasm or in the periplasmic region causes disruption of cellular function or disruption and disorganization of membranes^18^,^19^. It has been suggested that ZnO nanoparticles are able to slow down the growth of *E. coli* due to disorganization of *E. coli* membranes, which increases membrane permeability leading to accumulation of nanoparticles in the bacterial membrane and cytoplasmic regions of the cells^18^. A different protective mechanism of ZnO NPs has been suggested that ZnO NPs may protect intestinal cells from *E. coli* infection by inhibiting the adhesion and internalization of bacteria by preventing the increase of tight junction permeability and modulating cytokine^20^. Moreover, the electrostatic attraction between negatively charged bacterial cells and positively charged nanoparticles is crucial for the activity of nanoparticles as bactericidal materials. This interaction not only inhibits the bacterial growth but also induces the reactive oxygen species (ROS) generation, which leads to cell death^21^,^22^,^23^,^24^,^25^,^26^,^27^,^28^,^29^.

The superoxide radical, hydroxyl radical and hydrogen peroxide belonging to the ROS group can cause damage to DNA and cellular proteins, and may even lead to cell death^30^. Generally, nanoparticles with better photocatalytic activity have larger specific surface areas and smaller crystallite sizes, which increase oxygen vacancies, resulting in more ROS^31^,^43^. Earlier studies have proved that the terminal polar face (001) of ZnO NPs is more active than the nonpolar face (00–1) for photocatalytic H_2_O_2_ generation^31^,^32^.

Gram negative bacteria that produce enzymes called extended-spectrum beta-lactamases (ESBLs) are resistant to many penicillin and cephalosporin antibiotics and often to other types of antibiotics. The *Escherichia coli* (*E. coli*) and *Klebsiella pneumonia (K. pneumonia)* are predominant ESBL producers associated with urinary tract infection and sometimes it progresses to more serious infections like blood poisoning, which can be life threatening^33^,^34^,^35^,^36^. ESBLs that mediate resistance to third-generation cephalosporins are now observed worldwide in all species of Enterobacteriaceae, especially *Escherichia coli* and *Klebsiella pneumonia*^34^,^35^. Actually, ESBLs are the enzymes produced by gram-negative bacteria that have the ability to hydrolyze broad spectrum antibiotic containing an oximino group and are inhibited by β-lactamase inhibitors such as clavulanic acid, sulbactam and tazobactam^34^. Mustafa *et al.*^37^ and Ansari *et al.*^38^ have investigated the antibacterial potential of ZnO NPs against ESBLs producing *E. coli* and *K. pneumoniae*^37^,^38^. However, to the best of our knowledge, the antibacterial (ESBLs producing strains *E. coli* and *K. pneumoniae*) properties have not been reported for the Nd doped ZnO NPs.

In order to explore new strategies to identify and develop the next generation of drugs or agents to control bacterial infections, the antibacterial (ESBLs producing strains *E. coli* and *K. pneumoniae*) properties of the ZnO and Nd doped ZnO NPs are examined with the support of the structural and optical characterization studies.

## Results and Discussion

### X-ray diffraction studies

**Figure 1:**
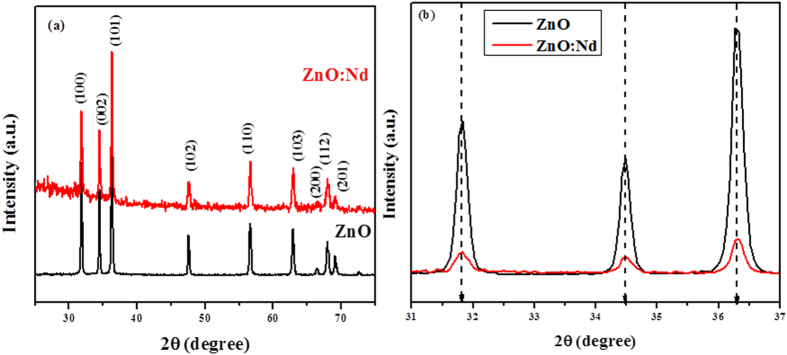
X-ray powder diffraction patterns of (**a**) pure ZnO and Nd doped ZnO NPs and (**b**) an enlarged version of the XRD pattern between 31° to 37°.

The X-ray diffraction peaks of pure ZnO and Nd doped ZnO NPs are shown in Fig. 1a. The pronounced diffraction peaks are clearly exhibiting the crystalline nature with peaks corresponding to (100), (002), (101), (102), (110), (103), (200), (112) and (201) planes. The standard diffraction peaks reveal that the crystal structure of ZnO and Nd doped ZnO NPs is of hexagonal wurtzite structure (space group p63mc, JCPDS data card no: 36–1451). Interestingly, the Nd doped ZnO NPs sample showed no additional phase formation. In order to examine the effect of Nd doping on the structure, an enlarged version of the XRD pattern between 31° to 37° is shown in Fig. 1b. It is worthy to mention that there is a slightly higher angle shift as compared to pure ZnO NPs, suggesting that Nd has been doped in ZnO in accordance with Vegard’s law. Since the ionic radii of Zn^2+^ and Nd^3+^ are 0.0740 nm and 0.0983 nm respectively^39^, the replacement of Nd^3+^ into Zn^2+^ sites makes the variation in ‘d’ values. The lattice constants ‘a’ and ‘c’ of wurtzite structure can be calculated by using the relation


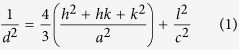


The lattice constant ‘a’ values are calculated as 3.24273 Å & 3.24274 Å and ‘c’ values are 5.20246 Å & 5.19914 Å for pure ZnO and Nd doped ZnO NPs respectively. The change in the lattice parameter values can be ascribed by the substitution of Nd^3+^ ion in Zn^2+^ sites, which has a higher ionic radius than Zn^2+^ in their tetrahedral coordinates.

The average crystallite size (D) of the NPs is calculated after appropriate background corrections from X-ray line broadening of the diffraction peaks using Debye Scherrer’s formula


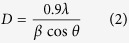


where λ is the wavelength of X-ray used (1.5405 Å), β is the angular peak width at half maximum in radians and θ is the Bragg’s diffraction angle. The average particle size is calculated as 47 nm and 33 nm for pure ZnO and Nd doped ZnO NPs respectively. The reduction in the particle size is mainly due to the distortion in the host ZnO lattice by the foreign impurity i.e., Nd^3+^.

The results and discussion are given in the Supporting Information (SI) for the following characterization studies that include FESEM, EDAX, FTIR and UV-Vis spectroscopy.

### Photoluminescence (PL) studies

**Figure 2:**
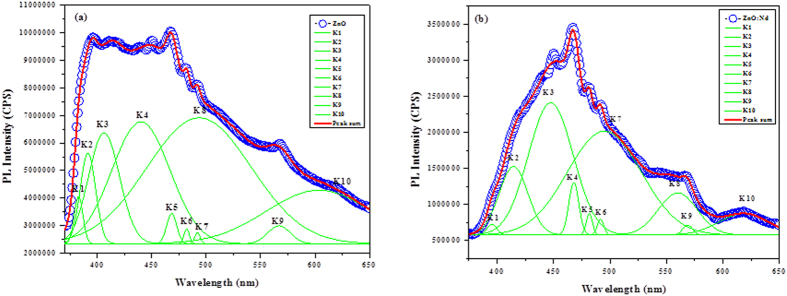
Gaussian de-composed photoluminescence emission spectra of (**a**) Pure ZnO NPs and (**b**) Nd doped ZnO NPs.

The Gaussian de-composed photoluminescence emission spectra of the as-synthesized pure and doped ZnO NPs samples recorded at the excitation wavelength of 350 nm are shown in Fig. 2(a,b). The PL emission is observed for both samples, from the very short wavelength of 375 nm to the longer wavelength of 650 nm. A good fit with ten peaks using a Gaussian function is obtained for the PL spectra of the samples, labeled as K1, K2, K3, K4, K5, K6, K7, K8, K9 and K10 (Fig. 2(a,b)). The solid lines represent the linear combination of the ten Gaussian peaks. K1 has the shortest wavelength and K10 has the longest wavelength.

The emission spectrum of the pure ZnO NP sample with ten peaks at 381 nm, 391 nm, 406 nm, 440 nm, 468 nm, 482 nm, 491 nm, 493 nm, 569 and 604 nm is shown in Fig. 2(a). The two near band edge emissions, violet emission, two blue emissions, three blue-green emissions, yellow emission and red emission are located at (381 nm & 391 nm), 406 nm, (440 nm & 468 nm), (482 nm, 491 nm & 493 nm), 569 nm, and 604 nm respectively. In the present case, the near band emissions (K1 & K2) are located in the UV region (381 nm & 391 nm) for the pure ZnO NPs. This UV peak corresponds to the radiative recombination of the free exciton–exciton collision process in the ZnO NPs.

The K3 peak is the origin of the violet emission centered at 406 nm and is ascribed to an electron transition from a shallow donor level of the natural zinc interstitials to the top level of the valence band^40^. The blue emission bands (K4 & K5) at 440 nm & 468 nm are attributed to singly ionized Zn vacancies^41^. There are three blue-green emission bands (K6, K7 & K8) at 482 nm, 491 nm and 493 nm due to a surface defects in the ZnO NPs corresponding to the transition between oxygen vacancy and oxygen interstitial defect. The yellow emission band (K9) is at 569 nm due to the presence of interstitial oxygen vacancies^42^. The K10 peak is the origin of the red emission centered at 604 nm and is attributed to zinc vacancies^43^.

As compared to pure ZnO NPs, the red shift is observed for the Nd doped ZnO NPs. The red shift may be from the different origins, such as electron phonon coupling, lattice distortion, localization of charge carriers due to interface effects and point defects.

The near band emission (K2) at 391 nm and violet emission (K3) at 406 nm observed in the pure ZnO NPs shift to violet band emission (K2) and blue emission (K3) at 414 nm and at 447 nm respectively in the Nd doped ZnO NPs. The violet emission and the enhanced blue emission for the rare earth Nd doped ZnO NPs sample should be come from the expanded Zn interstitial defect, which is attributed to the charge transfer process from the metal to the defect centers.

The band (K5) of pure ZnO NPs is observed as blue emission whereas the emission shifts into the blue-green emission in the case of Nd doped ZnO NPs due to a transition between the oxygen vacancy and interstitial oxygen and/or lattice defects related to oxygen and Zn vacancies.

For the Nd doped ZnO NPs, the blue-green band emission values (K6 and K7) shift from 482 nm to 491 nm and from 491 to 494 nm respectively. The band (K8) blue-green emission at 493 nm shifts to the yellow emission at 559 nm. The increasing band values for K6, K7 and K8 show an enhancement of oxygen vacancies. The changes in the emission values confirm the substitution of Nd^3+^ into the ZnO lattice sites.

### Antibacterial activity

#### Bacterial resistance and ESBL typing

**Figure 3:**
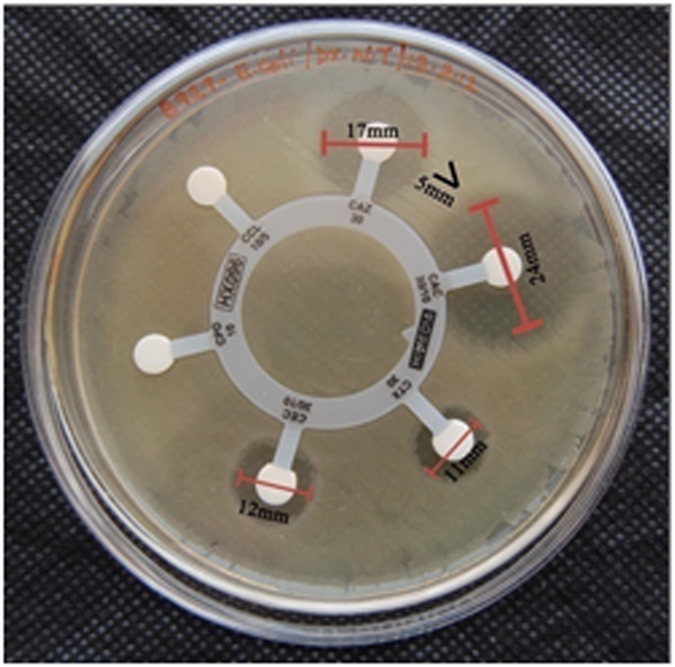
Detection of ESBL producers by combination disc method.

**Figure 4:**
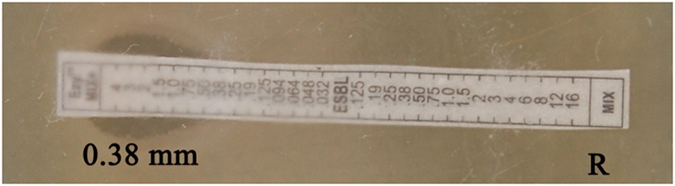
E-Test by triple detection strip- clear cut ESBL positive ceftazidime, cefotaxime, cefepime (Mix)/ceftazidime, cefotaxime, cefepime and clavulanic acid (Mix^+^) MIC value R/0.38 mm = >8 mm.

The two isolates tested for their resistance pattern in this *E. coli* were susceptible to novobiocin, rifampicin and erythromycin, which affirmed its resistance to remaining tested antibiotics. The strain *K. pneumonia* showed 100% of resistance to all the tested antibiotics and was susceptible only to amikacin. Applying CLSI ESBL typing of *E. coli* and *K. pneumonia* (Table 1), in the combination disc method showed that the isolates had “positive” result (Fig. 3). According to CLSI ESBL typing^44^ (triple detection strip), MIC criteria showed similar results (Fig. 4). Instead of cepfodoxime, fourth generation cepefime was used in the strip test as a marker for high level ESBL production.

**Figure 5:**
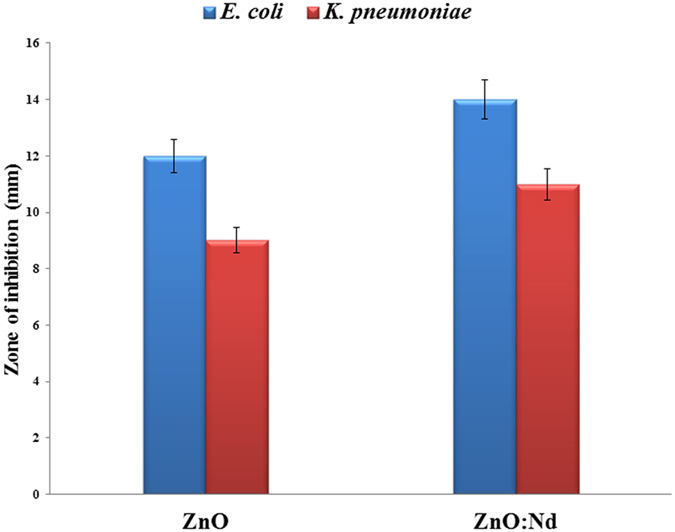
The size of the zone of inhibition formed around each disc, loaded with test samples, indicating the antibacterial activity towards ESBLs producing *E. coli* and *K. pneumoniae* for pure ZnO and Nd doped ZnO NPs.

Figure 5 shows the size of the zone of inhibition and antibacterial activity formed around the ZnO and Nd doped ZnO NPs loaded with test samples. The ZnO and Nd doped ZnO NPs exhibited the antibacterial activity. The antibacterial activity of the pure and doped ZnO NPs is explained as follows.

As mentioned earlier, the greater number of ROS is mainly attributed to the small crystallite size of the NPs, an increase in oxygen vacancies and the diffusion ability of the reactant molecules. In the present investigation, the antibacterial effect of the ZnO NPs samples is mainly due to the combination of various factors such as ROS and the release of Zn^2+^.

The first reason is the generation of hydrogen peroxide (H_2_O_2_) from the ZnO surface. The generation of highly reactive species such as OH^−^, H_2_O_2_ and O_2_^2−^ is explained below. Since ZnO NPs with defects can be activated by UV-visible light, electron-hole pairs (e^−^ h^+^) are created. The holes split H_2_O molecules (from the suspension of ZnO) into OH^−^ and H^+^. Dissolved oxygen molecules are transformed to superoxide radical anions (^•^O_2_^−^), which in turn react with H^+^ to generate (HO_2_^•^) radicals, which upon subsequent collision with electrons produce hydrogen peroxide anions (HO_2_^−^). They then react with hydrogen ions to produce molecules of H_2_O_2_. The generated H_2_O_2_ can penetrate into the cell membrane and kills the bacteria^45^.

The Nd doped ZnO NPs show the highest antibacterial activity. In case of biocidal activity, during the process, Nd^3+^ is not released and it cannot be responsible for the biocidal activity of the Nd doped ZnO NPs samples. However, the replacement of Nd in Zn sites enhances the photoactivity of the ZnO NPs. The smaller sized nanoparticles indeed have higher antibacterial activity^21^. Tong *et al.* reported that an appropriate crystallite size (ca. 33 nm) caused more antibacterial effects^46^. From the XRD patterns, the crystallite size of undoped and Nd doped ZnO NPs are found to be 47 nm and 33 nm respectively. From the XRD pattern of the Nd doped ZnO NPs, no secondary peaks corresponding to Nd are observed. The ionic radii of Nd^3+^ (0.0983 nm) and Zn^2+^ (0.074 nm) allow significant replacement in either structure. Hence, the enhanced antibacterial properties in the Nd doped ZnO NPs are also due to the replacement of some Nd ions in the Zn lattice sites.

From the photoluminescence study, as compared to ZnO NPs, oxygen vacancies are increased in the Nd doped ZnO NPs. The wavelength of the blue-green band K7 is at 494 nm for the Nd doped ZnO NPs, whereas the wavelength of the emissions is at 491 nm for the pure ZnO NPs. This shows the increased number of oxygen vacancies and interstitial oxygen vacancies in the Nd-doped ZnO NPs. The band K8 corresponds to blue-green emission for undoped ZnO NPs, but in the case of Nd doped ZnO NPs, the band K8 shifts to green emission. The wavelength of the emission observed at 493 nm for the pure ZnO NPs shifts to 559 nm for Nd doped ZnO NPs. This shows the increasing impurity levels corresponding to the singly ionized oxygen vacancies in the Nd doped ZnO NPs, and the transitions between photoexcited holes and singly ionized oxygen vacancies. This shows the increased number of oxygen vacancies, interstitial oxygen vacancies and singly ionized oxygen vacancies in the Nd-doped ZnO NPs, leading to a higher number of ROS as compared to the ZnO NPs.

**Figure 6:**
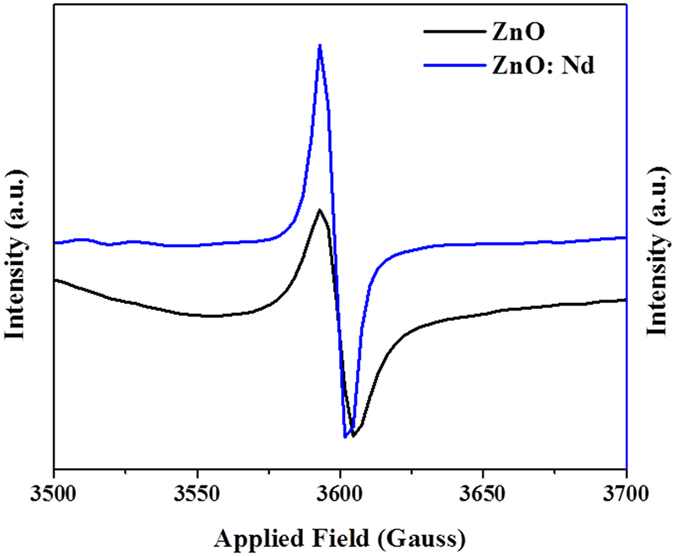
EPR spectra of ZnO and Nd doped ZnO NPs samples at room temperature.

EPR method is generally used to characterize the native defects in metal oxide NPs^47^. The room-temperature EPR spectra of the undoped and Nd-doped ZnO NPs are shown in Fig. 6. Singly ionized oxygen vacancy (V_o_) attributed to unpaired electrons trapped at oxygen vacancies is observed by EPR^16^,^48^. In the literature, the ZnO NPs samples with higher intensity of the signal are associated with more oxygen vacancies (V_o_) in it^28^,^49^. Therefore, according to Fig. 6, the pure ZnO NPs possess more intensity as compared to that of the Nd doped ZnO NPs. This result shows that the amount of oxygen vacancies in the Nd doped ZnO NPs is more than that in the pure ZnO NPs, which coincides with the results determined by the PL spectra also.

The NPs with uneven surfaces and rough edges have been found to adhere to the bacterial cellwall and cause damage to the cell membrane^50^. In the literature, spherical aggregates, fusiform-shaped microrods, nanosheet based flowers, microrod composed flowers and nanopetal built flowers of ZnO NPs have been reported and as compared to the other morphologies of ZnO NPs, fusiform shaped microrods exhibited the highest antibacterial activity^46^. From the FESEM images (Supplementary Figure S1), it is clear that the Nd doped ZnO NPs showed uneven ridges at the outer surface which lead to the antibacterial activity, whereas the ZnO NPs have smooth surfaces.

**Figure 7:**
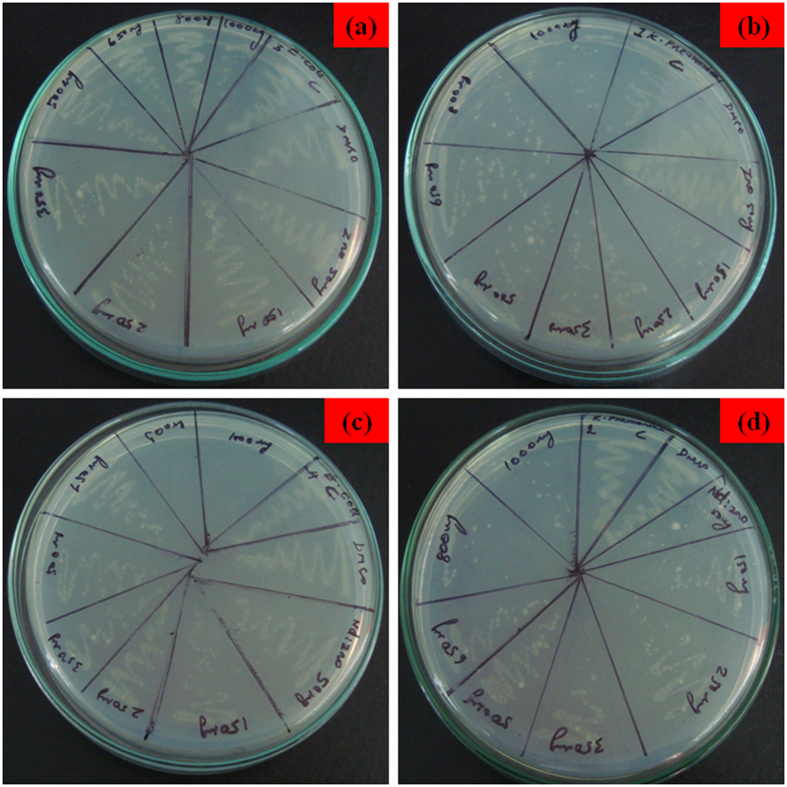
MBC results for the entire test against ESBLs producing (**a**) *E. coli* treated with ZnO NPs, (**b**) *K. pneumoniae* treated with ZnO NPs, (**c**) *E. coli* treated with Nd doped ZnO NPs (**d**) *K. pneumoniae* treated with Nd doped ZnO NPs.

Generally, bactericidal agents are much preferred in the clinical field because bactericides lead to rapid and better recovery from bacterial infections and also minimize the possibility of the emergence of drug resistance^51^. Since the undoped and Nd doped ZnO NPs exhibited relatively superior activity against all the pathogens, its minimum inhibitory concentration (MIC) and minimum bactericidal concentration (MBC) are ascertained. The MIC and MBC results for all of the test pathogens are shown in Fig. 7(a,d). Table 2 shows a comparison between present and reported MIC values of various metals doped ZnO NPs required to inhibit the growth of *E. coli*. Table 3 shows the MIC results for the entire test against ESBLs producing *E. coli* and *K. pneumoniae* for pure ZnO and Nd doped ZnO NPs. Table 3 showed that the control bacterial culture possessed 100% of cell growth. While increasing the concentration (50, 150, 250, 350, 500, 650, 800 and 1000 μg/mL) of ZnO and Nd doped ZnO NPs, the bacterial cell growth percentage decreased. The 800 μg/mL of Nd doped ZnO samples treated with *E. coli* strain resulted 100% of cell death as shown in Table 3. Thus, we conclude that from the present study, the minimal quantity of the Nd-doped ZnO NPs sample required to inhibit the bacterial growth is found to be 800 μg/mL for *E. coli*, which is mentioned in Table 2 for comparison.

#### Confocal laser scanning microscopic studies

**Figure 8:**
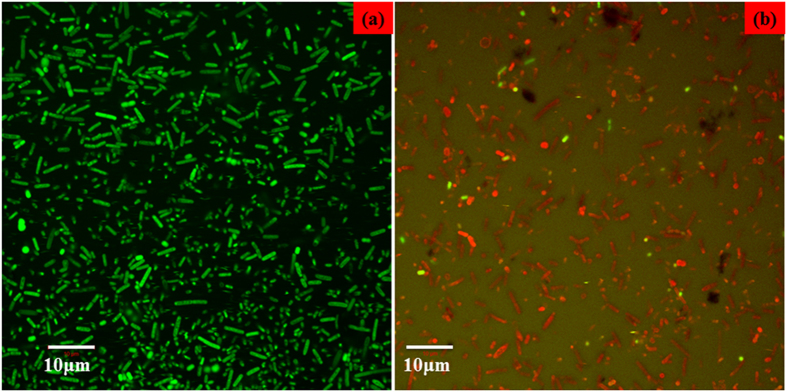
Confocal micrographs of ESBL producing *E. coli* (**a**) Control, (**b**) treated with 1000 μg/mL of Nd doped ZnO NPs.

**Figure 9:**
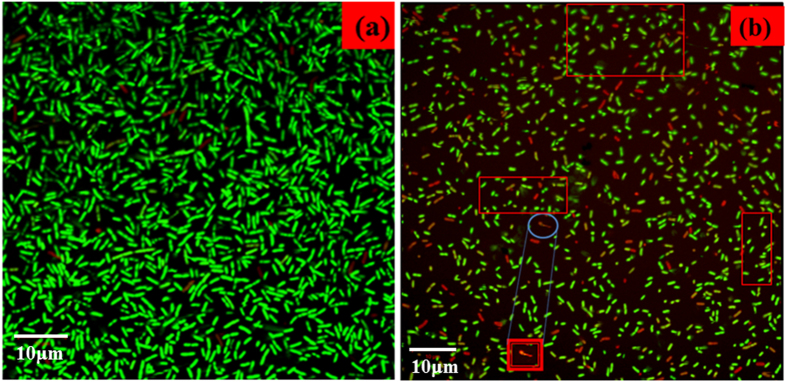
Confocal micrographs of ESBL producing *K. pneumoniae* (**a**) Control, (**b**) treated with 1000 μg/mL of Nd doped ZnO NPs.

The effect of Nd doped ZnO nanoparticles on the viability of *E. coli* and *K. pneumoniae* strains were studied by confocal laser scanning microscopy (CLSM) in the presence of Acridine orange/Ethidium Bromide (AO/EB) staining. It should be noted that in AO/ EB, Acridine orange stains both live and dead cells. Ethidium bromide stains only cells that have lost membrane integrity, i.e., EB permeates only cells which lost membrane integrity. Live cells appear as green in colour and dead cells appear as red in colour. The nanoparticles prepared in this study have the small particle sizes and thus cause the bacteria to coagulate in nanoparticle suspensions making it challenging to observe individual bacterium. Figures 8(a,b and 9a,b) represent dual-stained cells at magnification of 60X of *E. coli* and *K. pneumoniae* strains untreated (control) and treated with (1000 μg/mL) Nd doped ZnO NPs. The results from the dual staining suggest that the Nd doped ZnO NPs treated cells are dead as compared to untreated *E. coli* and *K. pneumoniae* cells. The majority of the untreated cells showed a green fluorescence due to the viable or live cells, indicating intact cell wall structure, whereas only a small percentage of the untreated cells showed red fluorescence denoting dead cells with non-permeable cell wall or membrane structure. Figure 8(b) in contrast, the cells (almost 99%) treated with 1000 μg/mL of Nd doped ZnO NPs exhibited red fluorescence indicating dead cells. These results suggest that the treatment of *E. coli* strain with the Nd doped ZnO NPs leads to cell death and/or bacteriostatic effect, which coincides with the results determined by MIC through optical density measurement. But *K. pneumoniae* cells treated with 1000 μg/mL of Nd doped ZnO NPs (Fig. 9b) show a more apoptotic nature as well as cell shrinkage, disorganization of both cell wall and cell membrane and dead cell of the bacteria.

#### Cell morphology by SEM imaging

The antibacterial actions are thought to be linked to interactions of the biocides with the cell membrane of the microorganisms. The agents then pierce into the cell and finally act at various target sites. Moreover, it is well accepted that the disorganization of the membrane by undesired or foreign substances can cause loss of the integrity of the membrane, which leads to malfunction of the permeability barrier. This ultimately causes the death of the cell^52^.

**Figure 10:**
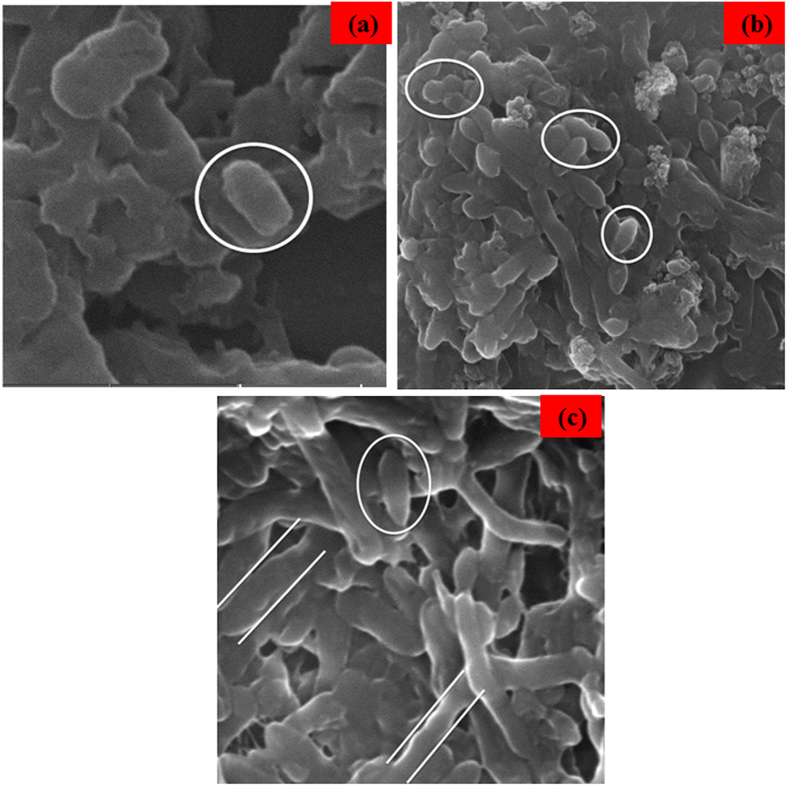
SEM image of (**a**) Control, (**b**) *E. coli* and (**c**) *K. pneumoniae* treated with MIC of Nd doped ZnO NPs slurries for 4 h.

Figure 10(a–c) shows that the activity of Nd doped ZnO NPs on the bacterial cell and both bacterial strains were examined using SEM analysis to look for structural changes in outer-membrane of the cells. Figure 10a shows the untreated control cell marked by a circle and these cells of bacterial species did not exhibit injury to the cell membrane. In the case of Nd doped ZnO NPs treated with *E. coli*, the cells are marked by the ellipses (Fig. 10b) whereas for the Nd doped ZnO NPs treated with *K. pneumoniae*, the cells are marked by parallel straight lines (Fig. 10c). The Nd doped ZnO NPs are on the surface of bacteria, leading to the disruption and disorganization of membranes. The bacterial loss of viability is correlated to an impairment of cell membrane integrity, which is highly consistent with the damage cell walls for both strains, revealed by the SEM analysis.

## Conclusion

In summary, the pure ZnO and Nd doped ZnO NPs were prepared by the co-precipitation method. The X-ray diffraction study confirmed that the prepared particles were of the hexagonal wurtzite structure. From the FESEM images, the pure and doped samples were found to exhibit nanorod and nanoflower like morphologies respectively. From the EDAX analysis, the chemical compositions were estimated for the prepared samples. From the recorded FT-IR spectra, the various vibrational frequencies were assigned for the pure ZnO and Nd doped ZnO NPs samples. The band gap of ZnO and Nd doped ZnO NPs were estimated as 3.34 and 3.26 eV from the UV-Vis spectroscopic measurements. The photoluminescence studies showed that the doping with ZnO NPs altered the band emission due to zinc vacancies, oxygen vacancies and surface defects.

The Nd doped ZnO NPs showed the highest antibacterial activity. In case of biocidal activity, during the process Nd^3+^ was not released and it could not be responsible for the biocidal activity of the Nd doped ZnO samples. From the XRD patterns, the crystallite size of undoped and Nd doped ZnO nanoparticles were found to be 47 nm and 33 nm respectively. The smaller crystallite sizes with more specific surface area led to higher antibacterial activity. From the EPR spectra, the amount of oxygen vacancies in the Nd doped ZnO NPs was more than that in the pure ZnO NPs, which coincided with the results determined by the PL spectra. The control bacterial culture possessed 100% of cell growth. While increasing the concentration (50, 150, 250, 350, 500, 650, 800 and 1000 μg/mL) of ZnO and Nd doped ZnO NPs, the bacterial cell growth percentage decreased. The 100% of cell death was observed for 800 μg/mL of Nd doped ZnO NPs samples treated with *E. coli*. So, 800 μg/mL of the Nd-doped ZnO NPs sample was found as the minimal quantity required to inhibit the bacterial growth for *E. coli* strain after carrying out the experiment in triplicates for the reliability of the results. The CLSM images showed that the cells were apoptotic. The SEM analysis revealed the bacterial loss of viability due to an impairment of cell membrane integrity with the damage of cell walls for both strains.

The ZnO NPs received much attention for their potential application in cancer therapy. The ZnO NPs are used in cancer therapy and their inherent preferential cytotoxicity against cancer cells can be used for new anti-cancer agents. In the literature, it has been reported that ZnO NPs killed the human lung and liver cancer cells, human glioma cells, human myeloblastic leukemia cells, cancerous T cells and Human breast cancer with no toxicity to the normal cells. So, Nd doped ZnO NPs can be used for the anticancer treatment. In order to use ZnO NP *in vivo*, further studies will be performed for the toxic effect of ZnO NPs. In addition, the ZnO NPs can be good candidates as antibacterial agents for external use in ointments, cosmetics etc. and can be used as drug carriers.

## Materials and Methods

### Chemicals and synthesis

The following high purity chemicals such as Zinc (II) nitrate hexahydrate (Zn (NO_3_)_2_ . 6H_2_O), Neodymium (III) nitrate hexahydrate (Nd (NO_3_)_3_ . 6H_2_O) and Sodium hydroxide (NaOH) were used as the precursors without further purification. The experimental procedures for the preparation of pure ZnO and Nd doped ZnO NPs samples are as follows:

For the preparation of pure ZnO NPs, 0.1 M of Zinc nitrate hexahydrate and 0.8 M of NaOH were separately dissolved in each 200 ml of distilled water using two 250 ml beakers. Then, NaOH solution was added drop wise to the Zinc nitrate solution which yielded a white precipitate. The solution with the white precipitate was stirred at room temperature for 6 h. This solution was refluxed for 24 h. Then, a clear solution was obtained, which found to be stable at ambient condition. Thereafter, the solution was washed several times with double distilled water and ethanol. Finally, the precipitate was dried at 120 °C. Thus, ZnO nanopowder was obtained.

Similarly, for the preparation of Nd doped ZnO NPs, 0.003 M of aqueous Neodymium nitrate hexahydrate solution was added into 0.097 M of the aqueous Zinc nitrate solution. 0.8 M of aqueous NaOH solution was added drop by drop to this homogenous mixture to get a white precipitate. The solution with the white precipitate was processed as said above to obtain Nd doped ZnO NPs sample. Thus, pure ZnO and Nd doped ZnO NPs samples were obtained. These samples were annealed at 700 °C for 5 h. The annealed samples were used for further analysis.

### Bacteria and resistance pattern

The bacterial strains *E. coli* and *K. pneumoniae* were made available from Medwin Hospital, Nampalli, Hyderabad in India. The resistance patterns of these bacterial strains were determined using the antibiotics ceftazidime (CAZ 30 mcg), cepfodoxime (CEP 10 mcg), amoxicillin (AM 30 mcg), novobiocin (NV 30 mcg), rifampicin (RIF 5 mcg), erythromycin (E 15 mcg), amikacin (AK 30 mcg), methicillin (MET 5 mcg), vancomycin (VA 30 mcg) and penicillin (P 10 units) adopting disk diffusion test (Himedia, India).

### Detection of ESBLs (HEXA G- minus 24 and E-test triple detection strip)

Phenotypic confirmation was done on the *E. coli* and *K. pneumoniae* by combination disc (Hexa disc) and the interpretations were recorded according to Clinical Laboratory Standard Institute (CLSI) guidelines. The indicators used were ceftazidime (30 mg), cefotaxime (30 mcg) and cepfodoxime (10 mcg) alone and in combination with clavulanic acid (10 mcg) separately (CLSI, 2012)^44^. Further, the ESBLs production of two isolates was confirmed using E-Test triple detection strip calibrated with MIC reading scales in μg/ml (Table 1). Positive ESBL was recorded when an enhanced inhibition zone of cephalosporin/clavulanic acid was >5 mm (combination method) and >8 mm (E-test) rather than cephalosporin alone.

The experimental methods are given in the Supporting Information (SI) which includes the preparation of test samples and bacterial cultures, determination of MIC and MBC. The experimental procedure employed for the confocal laser scanning microscopic studies, scanning electron microscopic investigation of bacteria and characterization techniques has also been included in the Supporting Information.

## Additional Information

**How to cite this article**: Hameed, A. S. H. *et al.* *In vitro* antibacterial activity of ZnO and Nd doped ZnO nanoparticles against ESBL producing *Escherichia coli* and *Klebsiella pneumoniae*. *Sci. Rep.* **6**, 24312; doi: 10.1038/srep24312 (2016).

## Supplementary Material

Supplementary Information

## Figures and Tables

**Table 1:** ESBL typing of *E. coli* and *K. pneumoniae* by combination disc and E-test triple detection methods.

Clinical isolates	Combination disc^a^	E-test by Enzymatic strip^b^	
	Ceftazidime, cefotaxime, cefpodoxime alone and combination with clavulanic acid	Ceftazidime, cefotaxime, cefepime and clavulanic acid (Mix^+^) MIC value	Ceftazidime, cefotaxime, cefepime (Mix) MIC value
*E. coli* (U655)	Positive (CAZ & CTX)	0.38 mm	R
*K. pneumoniae* (U759)	Positive (CAZ)	0.25 mm	R

R - No Zone.

^a^Ceftazidime (CAZ), cefotaxime (CTX) and cepfodoxime (CEP) (beta lactam) & ceftazidime, cefotaxime and cepfodoxime/clavulanic acid (beta lactam inhibitor) ESBL production is confirmed if a ≥5 mm increase in a zone diameter for one antimicrobial agent tested in combination with clavulanic acid versus its zone when tested alone.

^b^ESBL E-test positive MIC value of MIX (ceftazidime, cefotaxime, cefepime)/MIX^+^ (ceftazidime, cefotaxime, cefepime and clavulanic acid) = >8 mm as ESBL positive.

**Table 2:** Comparative MIC values of various metals doped ZnO NPs required to inhibit the growth of *E. coli*.

Sample	Minimum Inhibitory Concentration (MIC)
**ZnO:Nd (present study)**	800 (μg/mL)
**ZnO:La**^53^	25 (mg/mL)
**ZnO:Mg**^53^	25 (mg/mL)
**ZnO:Ag**^54^	512 (μg/mL)
**ZnO:Ag**^55^	600 (μg/mL)

**Table 3:** MIC results* for the entire test against ESBLs producing *E. coli* and *K. pneumoniae* for pure ZnO and Nd doped ZnO NPs.

Sample	Control	DMSO	50 μg/ml	150 μg/ml	250 μg/ml	350 μg/ml	500 μg/ml	650 μg/ml	800 μg/ml	1000 μg/ml
ZnO (*E. coli*)	0.615 ± 000	0.614 ± 090	0.59 ± 014	0.56 ± 020	0.472 ± 030	0.379 ± 041	0.299 ± 025	0.165 ± 020	0.078 ± 005	0.019 ± 003
ZnO (*K. pneumoniae*)	0.631 ± 010	0.629 ± 014	0.609 ± 005	0.581 ± 010	0.521 ± 0100	0.472 ± 010	0.385 ± 007	0.262 ± 004	0.142 ± 004	0.065 ± 004
ZnO:Nd (*E. coli*)	0.615 ± 000	0.614 ± 010	0.575 ± 012	0.543 ± 010	0.449 ± 005	0.315 ± 004	0.201 ± 005	0.091 ± 003	0 ± 000	0 ± 000
ZnO:Nd (*K. pneumoniae*)	0.631 ± 000	0.629 ± 007	0.595 ± 007	0.565 ± 010	0.495 ± 010	0.434 ± 010	0.325 ± 007	0.231 ± 007	0.106 ± 050	0.035 ± 005

^*^The anti-bacterial experiments were conducted in triplicates.
