# Author Correction: *In vitro* antibacterial activity of ZnO and Nd doped ZnO nanoparticles against ESBL producing *Escherichia coli* and *Klebsiella pneumoniae*

**DOI:** 10.1038/s41598-025-03233-y

**Published:** 2025-05-30

**Authors:** Abdulrahman Syedahamed Haja Hameed, Chandrasekaran Karthikeyan, Abdulazees Parveez Ahamed, Nooruddin Thajuddin, Naiyf S. Alharbi, Sulaiman Ali Alharbi, Ganasan Ravi

**Affiliations:** 1 PG and Research Department of Physics, Jamal Mohamed College, Tiruchirappalli, Tamil Nadu 620020 India; 2 Division of Microbial Biodiversity and Bioenergy, Department of Microbiology, Bharathidasan University, Tiruchirappalli, Tamil Nadu 600024 India; 3 Department of Botany and Microbiology, College of Science, King Saud University, Riyadh, 11451 Kingdom of Saudi Arabia; 4 School of Physics, Alagappa University, Karaikudi, Tamil Nadu 630004 India

Correction to: *Scientific Reports* 10.1038/srep24312, published online 13 April 2016

This Article contains errors in Table 3, where the standard deviation values are missing decimal points. The correct and incorrect values appear below.

Incorrect:

**Table:** 

Sample	Control	DMSO	50 μg/ml	150 μg/ml	250 μg/ml	350 μg/ml	500 μg/ml	650 μg/ml	800 μg/ml	1000 μg/ml
ZnO (E. coli)	0.615 ± 000	0.614 ± 090	0.59 ± 014	0.56 ± 020	0.472 ± 030	0.379 ± 041	0.299 ± 025	0.165 ± 020	0.078 ± 005	0.019 ± 003
ZnO (K. pneumoniae)	0.631 ± 010	0.629 ± 014	0.609 ± 005	0.581 ± 010	0.521 ± 0100	0.472 ± 010	0.385 ± 007	0.262 ± 004	0.142 ± 004	0.065 ± 004
ZnO:Nd (E. coli)	0.615 ± 000	0.614 ± 010	0.575 ± 012	0.543 ± 010	0.449 ± 005	0.315 ± 004	0.201 ± 005	0.091 ± 003	0 ± 000	0 ± 000
ZnO:Nd (K. pneumoniae)	0.631 ± 000	0.629 ± 007	0.595 ± 007	0.565 ± 010	0.495 ± 010	0.434 ± 010	0.325 ± 007	0.231 ± 007	0.106 ± 050	0.035 ± 005

Correct:

**Table:** 

Sample	Control	DMSO	50 μg/ml	150 μg/ml	250 μg/ml	350 μg/ml	500 μg/ml	650 μg/ml	800 μg/ml	1000 μg/ml
ZnO (E. coli)	0.615 ± 0.000	0.614 ± 0.090	0.59 ± 0.014	0.56 ± 0.020	0.472 ± 0.030	0.379 ± 0.041	0.299 ± 0.025	0.165 ± 0.020	0.078 ± 0.005	0.019 ± 0.003
ZnO (K. pneumoniae)	0.631 ± 0.010	0.629 ± 0.014	0.609 ± 0.005	0.581 ± 0.010	0.521 ± 0.0100	0.472 ± 0.010	0.385 ± 0.007	0.262 ± 0.004	0.142 ± 0.004	0.065 ± 0.004
ZnO:Nd (E. coli)	0.615 ± 0.000	0.614 ± 0.10	0.575 ± 0.0012	0.543 ± 0.010	0.449 ± 0.005	0.315 ± 0.004	0.201 ± 0.005	0.091 ± 0.003	0 ± 0.000	0 ± 0.000
ZnO:Nd (K. pneumoniae)	0.631 ± 0.000	0.629 ± 0.07	0.595 ± 0.007	0.565 ± 0.010	0.495 ± 0.010	0.434 ± 0.010	0.325 ± 0.007	0.231 ± 0.007	0.106 ± 0.050	0.035 ± 0.005

