# Supplementary material for: In vitro antibacterial activity of ZnO and Nd doped ZnO nanoparticles against ESBL producing Escherichia coli and Klebsiella pneumoniae
Source: Sci Rep. 2016 Apr 13;6:24312. doi: 10.1038/srep24312 (PMC4829841; doi:10.1038/srep24312)
Supplement: Supplementary Information [file srep24312-s1.pdf]

Supporting Information for

In vitro antibacterial activity of ZnO and Nd doped ZnO nanoparticles against ESBL producing *Escherichia coli* and *Klebsiella pneumoniae*

Abdulrahman Syedahamed Haja Hameed[a,]*, Chandrasekaran. Karthikeyan[a], Abdulazees Parveez Ahamed[b], Nooruddin Thajuddin[b], Naiyf S. Alharbi[d], Sulaiman Ali Alharbi[d] and Ganasan Ravi[c]

[a] *PG and Research Department of Physics, Jamal Mohamed College, Tiruchirappalli-620 020,Tamil Nadu, India.*

[b] *Division of Microbial Biodiversity and Bioenergy, Department of Microbiology, Bharathidasan University, Tiruchirappalli-600 024, Tamil Nadu, India.*

[c] *School of Physics, Alagappa University, Karaikudi-630004, Tamil Nadu, India.*

[d] *Department of Botany and Microbiology, College of Science, King Saud University, Riyadh, Kingdom of Saudi Arabia*

*Corresponding Author: Phone: +91-431 2331135/2332235; Fax: +91- 431 2331435; Email: hajahameed2001@gmail.com

Materials and methods

Preparation of test samples and bacterial cultures

All of the chemical reagents used in this study were analytical grade, obtained from Hi-Media Chemical Company, India. The test strains, *Escherichia coli* (U655) and *Klebsiella pneumoniae* (U759) were transferred into a tube containing 4 to 5 mL of nutrient broth and incubated at 35 ˚C until it achieved the turbidity of a 0.5 McFarland standard. Pure ZnO and Neodymium doped ZnO NPs samples were suspended in sterile dimethyl sulfoxide (DMSO) and constantly stirred until a uniform suspension was formed (10 mg/mL). The samples were photoactivated under visible light (2325–2500 lux) for 24 h.

Antibacterial assay

The antibacterial activity was determined by the disc diffusion method against the test bacteria on Muller-Hinton agar, according to the Clinical and Laboratory Standards Institute (CLSI) [1]. The media plates (MHA) were swabbed with bacteria 2-3 times by rotating the plate at 60˚ angles for each streak to ensure the homogeneous distribution of the inocula. After inoculation, discs (6 mm Hi-Media) loaded with 2 mg of the test samples were placed on the bacteria-seeded plates using sterile forceps. The plates were then incubated at 37 ˚C for 24 h. The inhibition zone around the discs was measured and recorded. DMSO served as the control, and assays were carried out in duplicates. We have simultaneously studied whether or not the DMSO without the ZnO NPs plays any active role as a biocide. We found that DMSO did not show any biocidal properties.

Determination of MIC and MBC

In the present study, the Minimum Inhibitory Concentration (MIC) and Minimum Bactericidal Concentration (MBC) were determined for ZnO and Nd-doped ZnO NPs by the agar dilution method. 2 mL of the test sample was added to 19 mL of molten nutrient agar and mixed adequately through mixing of the sample into the medium. The final concentrations of the sample in each plate were 150, 250, 350, 500, 650, 800 and 1000 µg/mL. Each plate was inoculated with test bacteria and the growth was determined by colony formation. The minimal quantity of the undoped ZnO and Nd doped ZnO NPs required for the bacterial activity was studied by adding 150, 250, 350, 500, 650, 800 and 1000 µg/mL concentrations of the nanoparticles into the bacterial culture and after 24 h, the Optical density values (600 nm) were measured. The lowest concentration, at which there was no regrowth of the bacteria, when

transferred from the test plate into new media, is called the MBC, and the MIC is the lowest concentration at which 99% of the bacterial growth was inhibited. The experiment was carried out in triplicates.

Confocal laser scanning microscopic studies

Apoptotic cell Acridine Orange/Ethidium Bromide (AO/EB) dual staining was used in our experiment. The collected bacteria were resuspended in 1 mL LB medium and then incubated with 10 μL AO/EB at 37 °C for 10 min (450-490 nm, and the final concentration was 5 μg/mL). Subsequently, the bacterial suspension was centrifuged at 5000 g for 5 min at 4 °C, and the supernatant was discarded. The unincorporated dyes were removed by washing with phosphate-buffered saline *(*PBS). One droplet of cell suspension (5 μL) was dropped on the freshly treated glass slide, and then it was covered with the coverslip without bubbles. The cells were microphotographed with the magnification of 60X using a laser scanning confocal microscope (Carl Zeiss 710, Zen Software 2011).

Microscopic investigation of bacteria

After the exposure, bacteria were fixed with 2% glutaraldehyde for 24 h. Samples were postfixed with 1% osmic acid for 2 h, and then fixed samples were dehydrated in an acetone gradient (35, 50, 70, 80, 95, and 100%) for 3 min, respectively. Finally, air-dried samples were analyzed by SEM (model: VEGA3 TESCAN).

Characterization techniques

The ZnO and Nd doped ZnO NPs were characterized by X-ray diffractometer (model: X'PERT PRO PANalytical). The diffraction patterns were recorded in the range of 20°-80° for

the ZnO samples where the monochromatic wavelength of 1.5405 Å was used. The samples were analyzed by FESEM (model: SUPRA 55) with EDAX (model: ULTRA 55). The FT-IR spectra were recorded in the range of 400-4000 cm^{-1} by using Shimadzu IR Affinity-1S spectrometer. The UV-Vis spectra were recorded in the wavelength range 190-1110nm by using Lambda 35 UV-Vis spectrophotometer. The photoluminescence (PL) measurement was performed on a (450W high pressure Xenon lamp as the excitation source, Photomultiplier, range 200-850 nm record for Horiba Jobin Yvon spectrofluorometer (model: FLUOROMAX-4). The electron paramagnetic resonance (EPR) measurement was conducted with a Bruker EMX Plus spectrometer using an X band (9.78 GHz) at room temperature.

Results and discussion

Morphology and Chemical Composition

The morphology and composition of as prepared nanoparticles are further investigated by FESEM and EDAX analyses. From **Fig. S1(a-b)**, the pure ZnO NPs look as a hexagonal nanorod like morphology with uniform grain boundaries and Nd doped ZnO NPs exhibit flower like morphology. The size reduction is due to the distortion in the host material incorporated with Nd^{3+}. The Nd^{3+} ions decrease the nucleation and subsequent growth rate of ZnO NPs.

The chemical composition of the pure ZnO and Nd doped ZnO NPs are found out using EDAX analysis. The typical EDAX spectra of pure ZnO and Nd doped ZnO NPs are shown in **Fig. S1(c-d)**. The doping concentration of Nd is found to be 2% in the case of Nd doped ZnO NPs.The atomic percentage of Zn and O are found to be 47% & 53% and 43% & 54% for the pure ZnO and Nd doped ZnO NPs samples respectively. From the EDAX analysis, the incorporation of Nd ions in ZnO is confirmed.

FT-IR spectroscopic studies

The FT-IR spectra of the prepared ZnO and Nd doped ZnO NPs are shown in **Fig. S2**. The FT-IR measurements are performed for the samples using the KBr pallet method in the wave number range 400-4000 cm^{-1}. The broad absorption in the frequency band 3750-3000 cm^{-1} is assigned to O-H stretching from residual alcohols, water and Zn-OH [2]. The absorption peak is observed at 3436 cm^{-1} for the ZnO NPs sample. The CO_2 peak is observed at 2369 cm^{-1} for the Nd doped ZnO samples. These CO_2 bands may arise due to some trapped CO_2 in air ambience [3]. The H-OH bending vibration is around 1640 and 1648 cm^{-1} for both pure and Nd doped ZnO samples. The stretching frequency of Zn-O is observed at 1497 cm^{-1} for the Nd doped ZnO NPs. It is noticed that the presence of the carbonated phase impurity has not been detected in the XRD pattern of the Nd doped ZnO samples. The Nd-O absorption peaks are obtained around 850-600 cm^{-1} [4]. From the FT-IR result, the Nd-O bands are observed at 848 cm^{-1} for the Nd doped ZnO NPs. The most intense broad absorption band at ~438 cm^{-1} is attributed to the stretching of vibration of ZnO [5]. The Zn-O stretching bands are observed at 422 and 451 cm^{-1} for the respective samples.

The vibration modes A_1 and E_1 of ZnO NPs are IR active. The infrared bands in the range 400-600 cm^{-1} are identified as E_1 (TO) at 435 cm^{-1} and the surface phonon modes SPM [A_1 (TO)] at 493 cm^{-1} and SPM [E_1 (TO)] at 540 cm^{-1} are observed [6]. A good fit with three peaks labeled as A1, A2 and A3 is obtained using a Gaussian function for the FT-IR spectra of undoped and Nd doped ZnO NPs. The fitting was carried out in the wave number range 400-600 cm^{-1} and is shown in **Fig. S3 (a-b)**.

The three peaks are observed at 428, 474 and 534 cm^{-1} for pure ZnO NPs sample. The peak A1 at 428 cm^{-1} can be assigned to [E_1 (TO)]. The interaction between electromagnetic (EM)

radiations and the particles depends on the size, shape and state of aggregation of the crystals [7]. Since the particle size in this study is much lower than the EM radiation, surface phonon modes (SPM) can be observed in the IR spectra. The SPM [A_1 (TO)] band (A2) around 473 cm^{-1} and [E_1 (TO)] band (A3) around 534 cm^{-1} can be identified as the SPM absorption in the IR spectra. As compared to undoped ZnO NPs, the entire IR modes shift to the blue side in the case of Nd doped ZnO NPs. The shift in the position of the bands can be correlated with their respective ionic radii as well as the structural changes due to the Nd doping in ZnO NPs. The values are given in **Table S1**.

UV- Vis-NIR spectroscopic studies

The absorbance of the samples depends on several factors such as band gap, oxygen deficiency, surface roughness and impurity centers [8]. The excitonic peaks are observed around 373 nm and 380 nm for the ZnO and Nd doped ZnO NPs respectively. The optical band gap energy of undoped and Nd doped ZnO NPs is calculated by the classical Tauc relation as given below [9].

$$\alpha h\upsilon = A(h\upsilon - E_g)^n$$

where E_g is the optical bandgap. A is a constant and the exponent n depends on the transition [10]. The value of n = 1/2, 3/2, 2, or 3 depends on the nature of the electronic transition (1/2 for allowed direct transition, 2 for allowed in-direct transition, 3/2 and 3 for forbidden direct and forbidden indirect transition, respectively). Considering a direct band transition in ZnO, a plot between $(\alpha h\upsilon)^2$ versus photon energy ($h\upsilon$) is drawn for ZnO and Nd doped ZnO NPs and is shown in **Fig. S4 (a-b)**. Extrapolation of the linear region of these plots to $(\alpha h\upsilon)^2 = 0$ gives the corresponding direct energy band gap. The band gap of 3.34 eV and 3.26 eV is obtained for the

ZnO and Nd doped ZnO NPs respectively. Comparing it with that of the pure ZnO NPs, the Nd doped ZnO NPs exhibit a red shift, where Nd doping results in a decrease of the optical band gap deviating from the Burstein-Moss shift [11-13]. The estimated bandgap values are close to that of the bulk ZnO. This gives a conclusion that there is no indication of the quantum size effect. The band gap of the Nd doped ZnO NPs shows non-montonic dependence on the Nd ions. This observation of non-monotonic behavior in the band gap of ZnO NPs as a function of Nd content strongly supports that Nd is indeed localized into the host lattice of ZnO.

Table S1 IR SPM modes of ZnO and Nd doped ZnO NPs.

Parameter modes	ZnO	ZnO:Nd
$[E_1(TO)]$ cm^{-1}	428	446
SPM $[A_1(TO)]$ cm^{-1}	474	516
SPM $[E_1(TO)]$ cm^{-1}	534	566

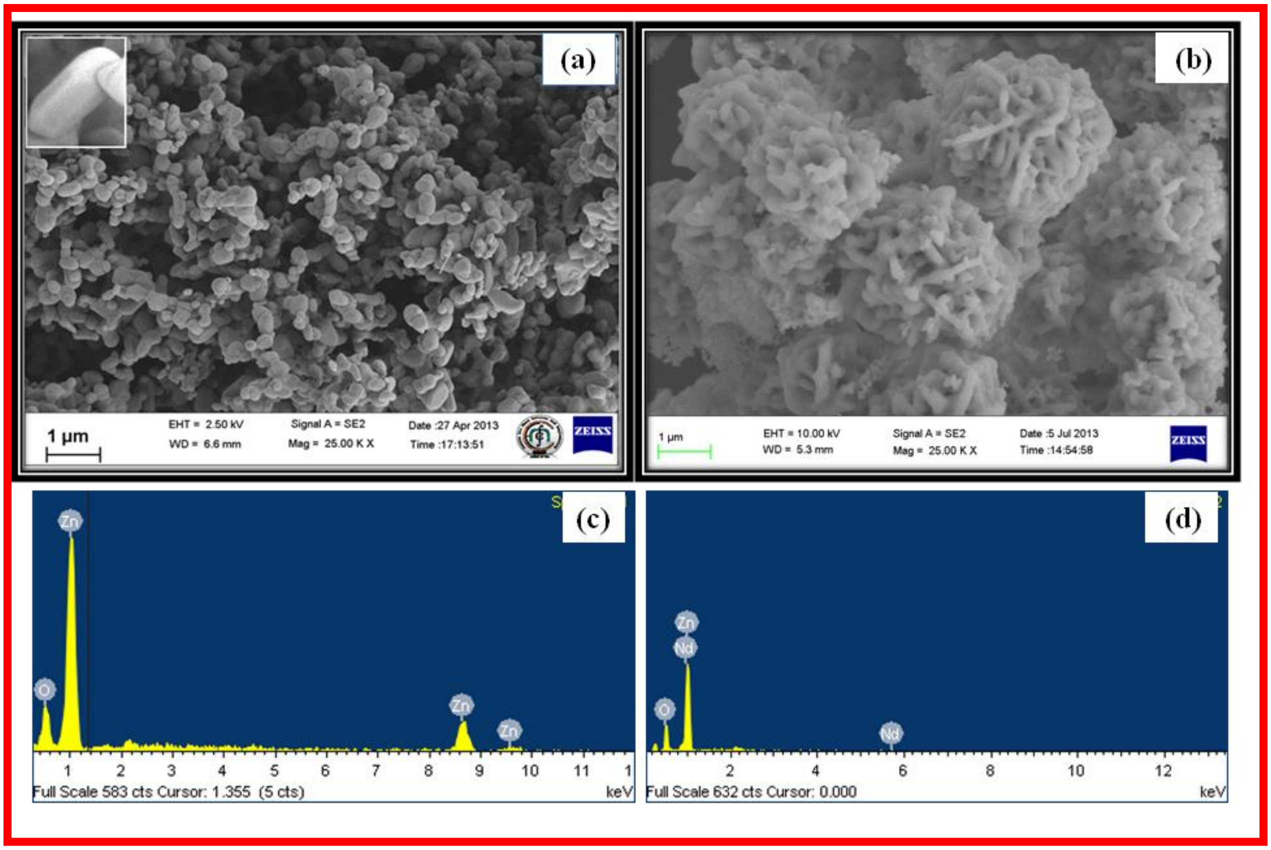

Figure S1 FESEM images of (a) pure ZnO NPs, (b) Nd doped ZnO NPs and EDAX spectra of (c) pure ZnO NPs (d) Nd doped ZnO NPs.

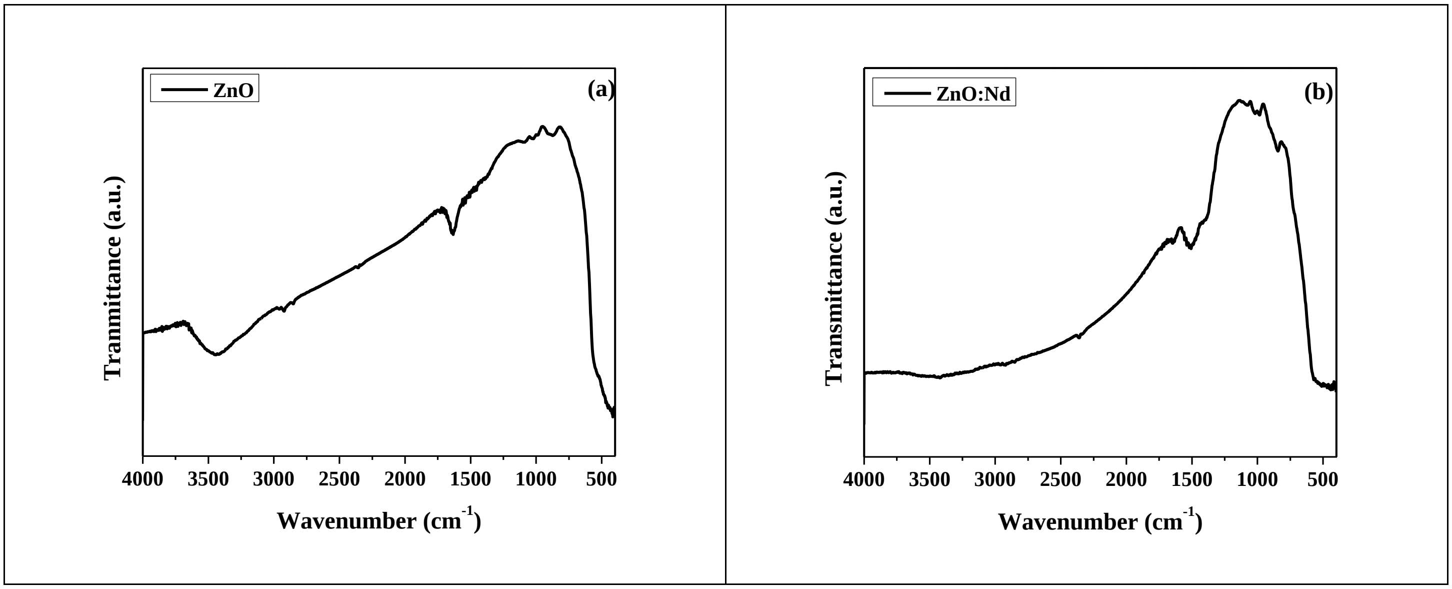

Figure S2 FTIR spectra of the pure ZnO and Nd-doped ZnO NPs.

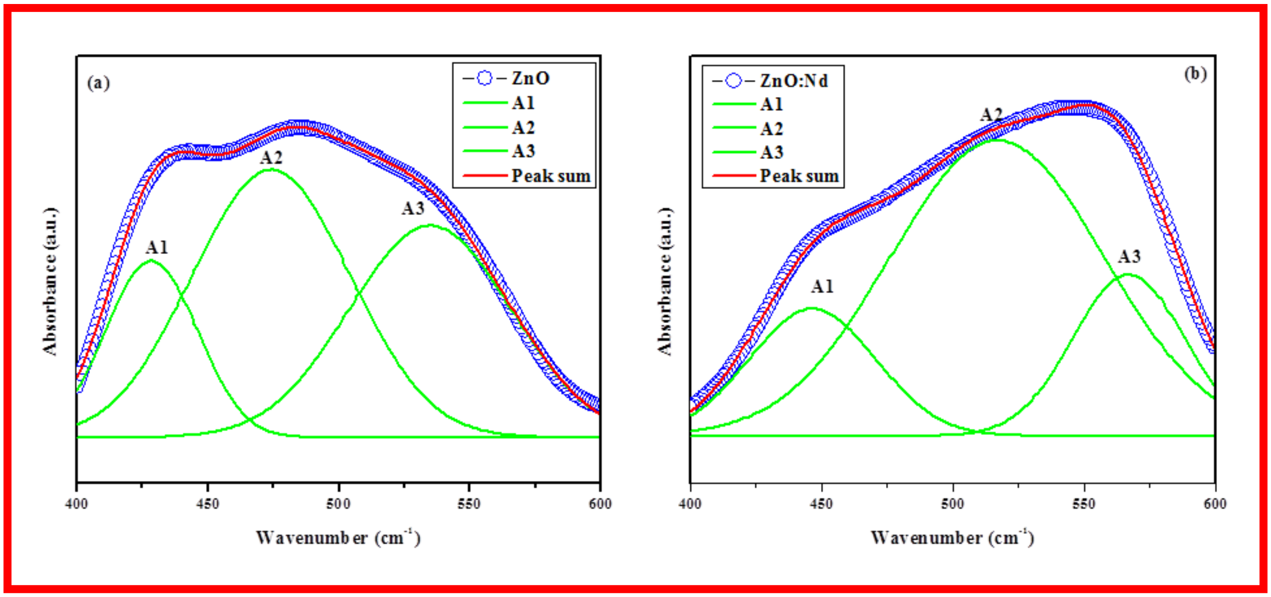

Figure S3 Fitted FT-IR absorption spectra of (a) ZnO NPs and (b) Nd doped ZnO NPs.

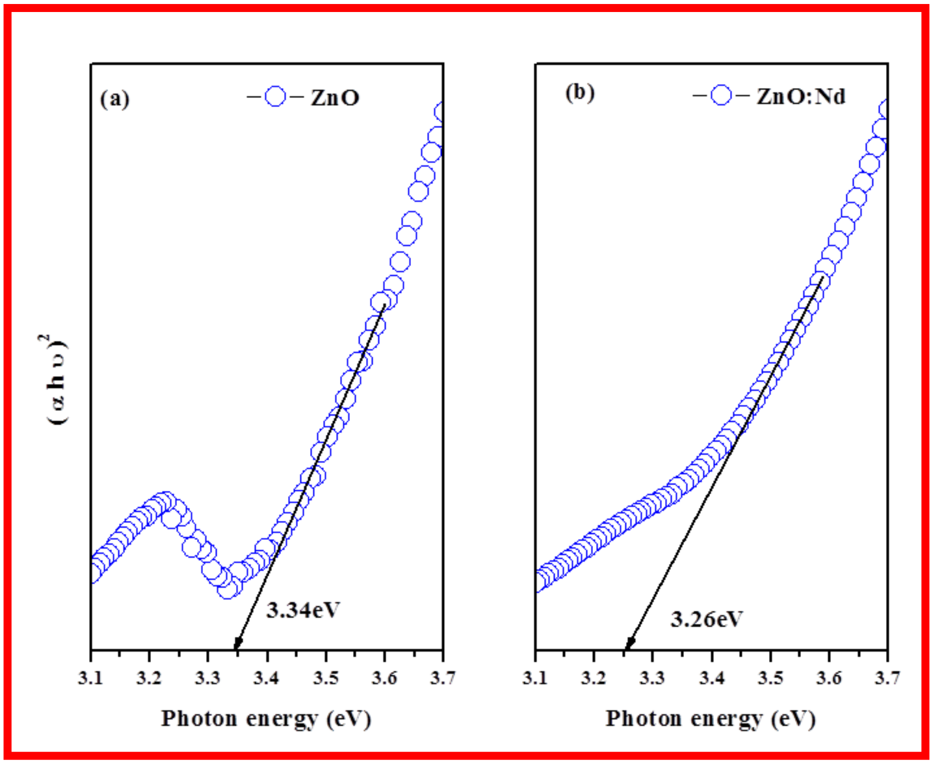

Figure S4 Band gap measurement for n = 1 excitonic state of (a) ZnO NPs and (b) Nd doped ZnO NPs.

References

1. Write, G. D. Resisting resistance: New chemical strategies for battling superbugs. *Chem. Bio.***7,** R127-R132 (2000).

2. Senthilkumar, S. et al. Influence of Mn doping on the microstructure and optical property of ZnO. *Mater. Sci. Semi. Process.* **11,** 6-12 (2008).

3. Oo, W. et al. Infrared spectroscopy of ZnO nanoparticles containing CO_2 impurities. *Appl. Phys. Lett.* **86,** 073111-1-073111-3 (2005).

4. Shahmoradi, B. et al, Modification of neodymium-doped ZnO hybrid nanoparticles under mild hydrothermal conditions. *Nanoscale* **2,** 1160-1164 (2010).

5. Xiong, G., Pal, U. & Serrano, J. G. Correlations among size, defects, and photoluminescence in ZnO nanoparticles. *J. Appl. Phys.* **101,** 24317-1-24317-6 (2007).

6. Ghosh, M., Dilawar, N. Bandyopadhyay, A. K. & Raychaudhuri, A. K. Phonon dynamics of Zn (Mg, Cd) O alloy nanostructures and their phase segregation. *J. Appl. Phys.* **106,** 084306-1-084306-6 (2009).

7. Cheng, B., Xiao, Y. Wu, G. & Zhang, L. The vibrational properties of one-dimensional ZnO:Ce nanostructures. *Appl. Phys. Lett.* **84,** 416-418 (2004).

8. Azam, A. et al. Study of electrical properties of nickel doped SnO2 ceramic nanoparticles. *J. Alloys Compd.* **506,** 237-242 (2010).

9. Tauc, J. in *Amorphous and liquid semiconductor*, P.159 (Plenum Press, 1974).

10. Xiao-Bo, L., Hong-Lie, S. Hui, Z. & Bin-bin, L. Optical properties of nanosized ZnO films prepared by sol-gel process. *Trans. Nonferrous Met. Soc*. China. **17,** S814-S817 (2007).

11. Lu, J. G. et al. Carrier concentration dependence of band gap shift in n-type ZnO: Al films. *J. Appl. Phys.* **101,** 083705-083707 (2007).

12. Benerjee, P. et al. Structural, electrical, and optical properties of atomic layer deposition Al-doped ZnO films. *J. Appl. Phys.* **108**, 043504-043507 (2010).

13. Yogamalar, N. R., & Bose, A. C. Absorption-emission study of hydrothermally grown Al:ZnO nanostructures. *J. Alloys Compd*. **509,** 8493-8500 (2011).
